# Deterioration of Cerebral Oxygenation by Aortic Arch Calcification Progression in Patients Undergoing Hemodialysis: A Cross-Sectional Study

**DOI:** 10.1155/2017/2852514

**Published:** 2017-10-03

**Authors:** Kiyonori Ito, Susumu Ookawara, Tomohisa Okochi, Yuichiro Ueda, Masaya Kofuji, Hideyuki Hayasaka, Takayuki Uchida, Haruhisa Miyazawa, Katsunori Yanai, Hiroki Ishii, Taisuke Kitano, Mitsutoshi Shindo, Keiji Hirai, Yoshio Kaku, Taro Hoshino, Osamu Tanaka, Kaoru Tabei, Yoshiyuki Morishita

**Affiliations:** ^1^Division of Nephrology, Department of Integrated Medicine, Saitama Medical Center, Jichi Medical University, Saitama, Japan; ^2^Division of Radiology, Department of Integrated Medicine, Saitama Medical Center, Jichi Medical University, Saitama, Japan; ^3^Department of Clinical Engineering, Saitama Medical Center, Jichi Medical University, Saitama, Japan; ^4^Minami-Uonuma City Hospital, Niigata, Japan

## Abstract

**Background:**

Near-infrared spectroscopy revealed that the regional saturation of oxygen (rSO_2_) in cerebral tissue is lower in hemodialysis (HD) patients than in healthy subjects. However, no study has examined the changes in cerebral oxygenation by aortic arch calcification (AAC) progression in HD patients.

**Methods:**

A total of 104 HD patients were divided into four groups by AAC grade determined using chest radiography: 23 patients at grade 0, 24 at grade 1, 30 at grade 2, and 27 at grade 3. Differences in clinical parameters, including cerebral rSO_2_, among AAC grades were investigated and atherosclerotic parameters affecting cerebral rSO_2_ values were identified.

**Results:**

Cerebral rSO_2_ significantly decreased as AAC progressed (AAC grade 3 versus grade 0, *p* < 0.01 versus grade 1, *p* < 0.05). Multivariate logistic regression analysis was performed using parameters with *p* values < 0.20 in univariate analysis between cerebral rSO_2_ values less than the mean and atherosclerotic parameters. AAC grades 2 and 3, serum phosphate level, and history of smoking were factors associated with the cerebral rSO_2_ decrease.

**Conclusions:**

Cerebral rSO_2_ significantly decreased as AAC progressed and was independently associated with higher AAC grade, serum phosphate level, and history of smoking.

## 1. Introduction

According to a 2012 year-end report, cerebrovascular disease is a main cause of death in patients receiving hemodialysis (HD) in Japan [1]. A lower estimated glomerular filtration rate is associated with lower cerebral blood flow [2], and patients with chronic kidney disease or who were receiving HD tend to develop cerebral infarction with high frequency even if they were asymptomatic [3, 4]. Therefore, it is important to recognize that patients with renal impairment may have vascular disorders, including ischemic stroke. Regarding the evaluation of vascular disorders, aortic arch calcification (AAC) using chest radiography has been a concern in clinical settings [5, 6]. To date, AAC was reportedly associated with cerebral ischemic disorder [7], in addition to cardiovascular disease [8].

Near-infrared spectroscopy (NIRS) has recently been used in various clinical settings to measure the regional saturation of oxygen (rSO_2_), a real-time marker of tissue oxygenation [9–14]. Cerebral rSO_2_ values were reportedly significantly lower in patients receiving HD than in healthy controls [15, 16]. Furthermore, these values were associated with pH, HD duration, serum albumin concentration, and the presence of diabetes mellitus [16]. However, no studies have examined the correlation between cerebral oxygenation and vascular disorders represented by AAC in HD patients. Therefore, here we aimed to clarify the associations between AAC and clinical parameters, including cerebral rSO_2_ values, in patients receiving HD.

## 2. Materials and Methods

We included patients receiving HD who met the following criteria: (1) with ESRD receiving intermittent HD, (2) AAC grade confirmed by chest radiography, and (3) presence of arteriovenous fistula as vascular access for HD. The exclusion criteria included any uncontrolled patients' condition: congestive heart failure with pleural effusion, acute cerebrovascular disease, obvious disturbing condition including cognitive impairment, and lung disease with shortness of breath at rest or high concentrated oxygen administration.

One hundred and four patients receiving HD were included (80 men and 24 women; mean age, 67.4 ± 9.6 years; mean HD duration, 4.7 ± 6.7 years). The causes of chronic renal failure were diabetes mellitus (51 patients), chronic glomerulonephritis (23 patients), nephrosclerosis (15 patients), and other (15 patients). Each patient was receiving maintenance HD two or three times a week with each HD session lasting 3 or 4 hours. The patients' general characteristics are summarized in Table 1. Written informed consent was obtained from all participants and this study was approved by the Institutional Review Board of Saitama Medical Center, Jichi Medical University, Japan (RIN 14–24), and conformed to the provisions of the Declaration of Helsinki (as revised in Tokyo in 2004).

### 2.1. Data Collection

#### 2.1.1. Patients' Baseline Characteristics and Clinical Laboratory Measurement

We collected the patients' baseline characteristics and other relevant data from their medical charts. The primary disease causing the dialysis requirement as well as any history of ischemic heart disease (IHD), cerebrovascular disease, or smoking, were recorded on the basis of medical records in our hospital.

Blood pressure (BP) and heart rate were measured with patients in the supine position before the HD session at the same time as the cerebral rSO_2_ measurement. Each patient's body mass index was calculated as weight (kg) divided by the square of height (m^2^) by each patient's dry weight. Blood samples were obtained at ambient temperature from the arteriovenous fistula of each patient prior to HD.

Chest radiography was performed within 1 month of cerebral rSO_2_ measurement, and the AAC grade was determined by two or more radiologists, as recommended in previous reports [5, 6]. We then examined the associations between AAC grade and clinical parameters, including cerebral rSO_2_ value, in each patient.

#### 2.1.2. Cerebral Oxygenation Monitoring

Cerebral rSO_2_ was monitored at the forehead using an INVOS 5100C saturation monitor (Covidien Japan, Tokyo, Japan) that utilizes NIRS technology. This instrument uses a light-emitting diode, which transmits near-infrared light at two wavelengths (735 nm and 810 nm), and two silicon photodiodes that act as light detectors to measure oxygenated Hb and deoxygenated Hb. The ratio of oxygenated Hb to total Hb (oxygenated Hb + deoxygenated Hb) signal strength, which is the corresponding percentage, is read as a single numerical value that represents the rSO_2_ [17, 18].

All data obtained using this instrument were immediately and automatically stored in sequence. The interobserver variance for this instrument, that is, reproducibility of the rSO_2_ measurement, is acceptable as previously reported [19–21]. Therefore, cerebral rSO_2_ is considered reliable for estimating the actual cerebral oxygenation. Furthermore, the light paths leading from the emitter to the different detectors share a common part: the 30 mm detector assesses superficial tissue, while the 40 mm detector is used to assess deep tissue. By analyzing the differential signals collected by the different detectors, the current data for cerebral rSO_2_ values were supposed to be obtained in deep tissue 20–30 mm from the body's surface [22, 23]. These measurements were performed at every 6-second interval during cerebral oxygenation monitoring.

Prior to undergoing HD, the included patients rested in the supine position for at least 10 minutes to reduce the influence of postural change. An rSO_2_ measurement sensor was attached to the patient's forehead for measurement in the resting state. Thereafter, rSO_2_ was measured for 5 min before HD, and we evaluated the mean rSO_2_ for 5 min, as a marker of cerebral oxygenation, in each patient.

#### 2.1.3. Inclusion of Atherosclerotic Parameters

In this study, we examined the relationship between cerebral rSO_2_ values and atherosclerotic parameters in addition to the severity of AAC grade, using univariate and multivariate logistic regression analysis. Atherosclerosis/arteriosclerosis was reportedly increased with cardiovascular risk factors, including advanced aging, smoking, hypertension, diabetes mellitus, dyslipidemia, abnormal calcium/phosphate metabolism, and inflammation [24, 25]. Therefore, we included several clinical parameters as atherosclerotic parameters in univariate and multivariate logistic regression analysis as follows: age, smoking, systolic and diastolic BP, serum calcium and serum phosphate concentration, high density lipoprotein cholesterol, low density lipoprotein cholesterol, triglyceride, blood glucose, hemoglobin A1c, C-reactive protein levels, and severity of AAC grade.

### 2.2. Statistical Analysis

Data are expressed as mean ± standard deviation. An unpaired Student's *t*-test was used to compare the two groups. One-way analysis of variance with Tukey's test was used to evaluate the significance among the four groups that were divided by AAC grade. A multivariate logistic regression analysis was performed to identify the atherosclerotic factors associated with a cerebral rSO_2_ value less than the mean using stepwise procedure. In this study, the cut-off value for cerebral rSO_2_ was 48.6%. The atherosclerotic parameters with a *p* value of 0.20 in univariate analysis were included in a stepwise manner. All analyses were performed using IBM SPSS Statistics for Windows, version 19.0. Differences with values of *p* < 0.05 were considered statistically significant.

## 3. Results

**Figure 1:**
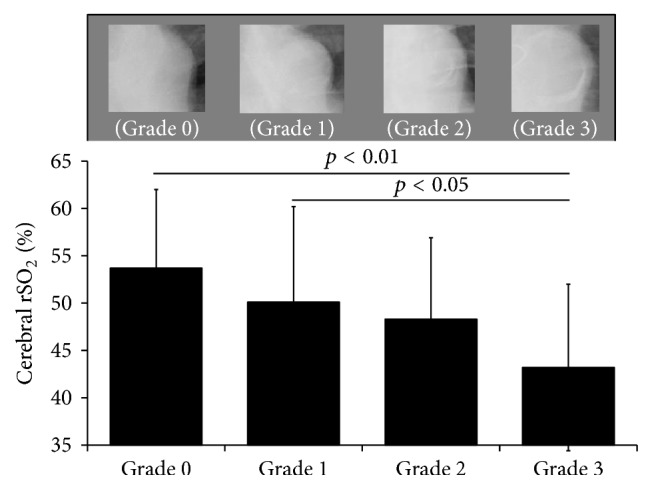
Association between AAC grade and cerebral rSO_2_ value. AAC, aortic arch calcification; rSO_2_, regional saturation of oxygen.

In this study, the mean cerebral rSO_2_ at rest in patients receiving HD was 48.6 ± 9.6%. Patients receiving HD were divided into four groups by AAC grade (from grade 0 to grade 3). There were 23 patients at grade 0, 24 patients at grade 1, 30 patients at grade 2, and 27 patients at grade 3. Table 1 shows the patients' characteristics in each group. Several factors differed significantly among these four groups: age, HD duration, primary disease causing HD requirement, history of IHD, smoking, albumin, and cerebral rSO_2_. In particular, as shown in Figure 1, cerebral rSO_2_ values decreased as AAC progressed, and these values at AAC grade 3 were significantly lower than those at AAC grades 0 and 1 (AAC grade 0, 53.7 ± 8.3%; grade 1, 50.1 ± 10.1%; grade 2, 48.3 ± 8.6%; grade 3, 43.2 ± 8.8%; AAC grade 3 versus grade 0, *p* < 0.01; AAC grade 3 versus grade 1, *p* < 0.05). Furthermore, multivariate logistic regression analysis was performed using the following atherosclerotic parameters: smoking, systolic BP, serum calcium, serum phosphate, low density lipoprotein cholesterol, blood glucose, C-reactive protein, and AAC grades 2 and 3 (*p* < 0.20 compared with cerebral rSO_2_ values less than mean monitored in this study in univariate analysis). As shown in Table 2, AAC grade 2 (odds ratio [OR] = 4.626, 95% confidence interval [CI] = 1.241–17.244) and AAC grade 3 (OR = 4.333, 95% CI = 1.134–16.551) in addition to serum phosphate (OR = 3.487, 95% CI = 1.340–9.090) and history of smoking (OR = 4.132, 95% CI = 1.414–12.048) were independently associated with cerebral rSO_2_ values less than the mean in this study.

## 4. Discussion

Previous reports in nondialysis patients have shown that AAC is related to the presence of diabetes mellitus and renal dysfunction [5] and a strong independent predictor of cardiovascular events, apart from the traditional risk factors [8]. On the other hand, in end-stage renal disease patients, vascular calcification, including AAC, is usually accelerated by mineral and bone disorder [26, 27] and associated with cardiovascular and all-cause mortality [26, 28–30]. Therefore, it is important to pay attention to the presence of vascular calcification, which is represented by AAC, in the clinical setting.

However, despite the large number of studies addressing the correlation between AAC and cardiovascular disease, few reports have examined the correlation between AAC and cerebrovascular disorders [7, 31]. Using magnetic resonance imaging on ischemic stroke patients not receiving dialysis, AAC was reportedly associated with cerebral small-artery occlusion and white matter disease [7]; therefore, such vascular disorders might directly influence the cerebral circulation. Furthermore, cerebral circulation dysregulation leads to a decrease in regional cerebral blood flow [32] and leukoaraiosis, an indicator of cerebral ischemia with a high prevalence in the anterior circulation of the brain, has also been reported as an independent risk factor for stroke [33] in dialysis patients. In the present study, decreased cerebral rSO_2_ values may have reflected a decreased oxygen supply in the brain via cerebral macro- and/or microcirculation impairments, which may explain the reason why AAC progression was negatively associated with cerebral rSO_2_ value.

The multivariate regression analysis in this study demonstrated that serum phosphate level, history of smoking, and higher AAC grade were independently associated with cerebral rSO_2_. Hyperphosphatemia impairs endothelial function by increasing reactive oxygen species and inhibiting endothelial nitric oxide synthase [34] and contributes to vascular calcification progression [30], which would lead to poor prognosis in patients receiving HD [35]. Thus, cerebral rSO_2_ might be affected by cerebral atherosclerosis progression induced by hyperphosphatemia, although chronic kidney disease -mineral and bone disorder was well-managed in the HD patients in this study. Smoking is a well-known major traditional factor of atherosclerosis. Furthermore, smoking itself reportedly injures endothelial cells [36, 37] and accelerates vascular atherosclerotic changes. Thus, this might be why a history of smoking was significantly associated with cerebral oxygenation in this study.

Cerebral rSO_2_ values were significantly associated with the higher AAC grade, which might indicate that vascular disease with AAC decreases cerebral rSO_2_ values. The presence of AAC was associated with arterial stiffness dysregulation [38] and carotid distensibility [39]. Furthermore, arterial stiffness disorders are related to endothelial dysfunction [40] and lead to cerebral circulation dysregulation [41]. Therefore, the presence of AAC might be associated with cerebral microcirculatory impairment via the arterial stiffness disorders, which may have led to the decreased cerebral rSO_2_ in this study.

This study had several limitations. First, the sample size was relatively small and the most of the patients were men. This study was also cross-sectional; therefore, it remains unclear whether the relationships confirmed in this study reflect causes or effects. Furthermore, AAC was only confirmed on the basis of chest radiography in each patient; therefore, elevated vascular calcification might not be accurate compared with other imaging examinations, including computed tomography and magnetic resonance angiography. Therefore, further studies are needed to clarify the associations between vascular calcification, including AAC, and cerebral oxygenation in patients receiving HD.

## 5. Conclusions

Cerebral rSO_2_ values significantly decreased as AAC progressed. Furthermore, cerebral rSO_2_ value was independently associated with a higher AAC grade in addition to serum phosphate level and history of smoking.

## Figures and Tables

**Table 1:** Baseline characteristics by AAC grade.

AAC grade	Grade 0	Grade 1	Grade 2	Grade 3
*N*	23	24	30	27
Sex (men), *n* (%)	18 (78)	20 (83)	21 (70)	21 (78)
Age, years	64 ± 11	64 ± 10	71 ± 8^c^	70 ± 7
HD duration, years	2.6 ± 5.7	5.0 ± 7.6	4.3 ± 7.5	6.7 ± 5.5
Primary disease, *n* (%)				
Diabetes mellitus	11 (48)	7 (29)	20 (70)^c^	13 (48)
Nephrosclerosis	2 (9)	4 (17)	4 (12)	5 (19)
Chronic glomerulonephritis	8 (34)	8 (33)	4 (12)	3 (11)
Others	2 (9)	5 (21)	2 (6)	6 (22)
Medical history, *n* (%)				
Ischemic heart disease	3 (13)	10 (42)	8 (28)	20 (74)^bd^
Cerebral infarction	4 (17)	8 (33)	4 (14)	4 (15)
Smoking	12 (52)	22 (92)^a^	17 (61)	20 (74)
Body mass index, kg/m^2^	22 ± 4	23 ± 3	22 ± 3	23 ± 3
Systolic BP, mmHg	145 ± 23	139 ± 18	150 ± 20	137 ± 23
Diastolic BP, mmHg	79 ± 15	78 ± 13	73 ± 13	69 ± 15
Pulse pressure, mmHg	66 ± 16	62 ± 18	77 ± 20^c^	69 ± 14
Heart rate, per min	76 ± 11	75 ± 15	71 ± 16	73 ± 12
Oxygen saturation, %	96 ± 1	95 ± 2	94 ± 5	95 ± 3

Medication, *n* (%)				

RAS blocker (ACEI and/or ARB)	12 (52)	15 (63)	17 (57)	14 (52)
Calcium channel blocker	14 (61)	15 (63)	22 (73)	15 (56)
Beta blocker	8 (35)	12 (50)	19 (63)	15 (56)
Vitamin D analog	11 (48)	10 (42)	13 (43)	11 (41)
Phosphate binder	15 (65)	13 (54)	19 (63)	18 (67)
Calcium-containing phosphate binder	14	10	15	13
Non-calcium-containing phosphate binder	4	7	10	13
Cinacalcet	1 (4)	1 (4)	3 (10)	4 (15)
Statin	5 (22)	8 (33)	10 (33)	12 (44)
Antiplatelet agents	8 (35)	11 (46)	13 (43)	19 (70)
Erythropoiesis stimulating agent	21 (91)	22 (92)	30 (100)	24 (89)

Albumin, g/dL	3.3 ± 0.6	3.2 ± 0.5	3.1 ± 0.5	2.9 ± 0.6^a^
Serum sodium, mEq/L	138 ± 4	137 ± 5	137 ± 3	137 ± 4
Serum potassium, mEq/L	4.4 ± 0.8	4.1 ± 0.8	4.2 ± 0.8	4.1 ± 0.5
Serum calcium, mg/dL	8.3 ± 0.7	8.9 ± 1.1	8.5 ± 1.1	8.8 ± 0.9
Serum phosphate, mg/dL	4.8 ± 1.2	4.4 ± 1.5	4.8 ± 1.3	4.4 ± 1.3
BUN, mg/dL	60 ± 14	55 ± 26	52 ± 20	51 ± 17
Cr, mg/dL	9.1 ± 2.2	8.3 ± 2.2	8.5 ± 2.4	8.2 ± 2.2
Hb, g/dL	9.9 ± 1.3	9.9 ± 1.6	9.4 ± 1.5	9.3 ± 1.5
Blood glucose, mg/dL	159 ± 73	149 ± 74	133 ± 37	152 ± 48
HbA1c, %	5.5 ± 1.0	5.5 ± 0.8	5.8 ± 1.0	5.7 ± 0.8
Total cholesterol, mg/dL	156 ± 32	148 ± 39	151 ± 40	149 ± 32
HDL cholesterol, mg/dL	47 ± 20	43 ± 16	45 ± 14	36 ± 14
Non-HDL cholesterol, mg/dL	109 ± 24	107 ± 29	105 ± 36	114 ± 29
LDL cholesterol, mg/dL	84 ± 23	77 ± 26	80 ± 31	88 ± 28
Triglyceride, mg/dL	96 ± 45	111 ± 33	100 ± 54	117 ± 57
C-reactive protein, mg/dL	0.7 ± 1.4	3.4 ± 8.8	1.2 ± 1.6	3.2 ± 4.9

Cerebral rSO_2_ value, %	53.7 ± 8.3	50.1 ± 10.1	48.3 ± 8.6	43.2 ± 8.8^bc^

AAC, aortic arch calcification; HD, hemodialysis; BP, blood pressure; RAS, renin-angiotensin system; ACEI, angiotensin-converting enzyme inhibitor; ARB, angiotensin II receptor blocker; BUN, blood urea nitrogen; Cr, creatinine; Hb, hemoglobin; HbA1c, hemoglobin A1c; HDL, high density lipoprotein; LDL, low density lipoprotein; rSO_2_, regional saturation of oxygen. ^a^Significant versus the AAC grade 0 group (*p* < 0.05). ^b^Significant versus the AAC grade 0 group (*p* < 0.01). ^c^Significant versus the AAC grade 1 group (*p* < 0.05). ^d^Significant versus the AAC grade 2 group (*p* < 0.01).

**Table 2:** Multivariate regression analysis for cerebral rSO_2_ value.

	Univariate analysis	*p* value	Multivariate analysis	*p* value
	Odds ratio (95% CI)		Odds ratio (95% CI)	
Age	1.095 (0.503–2.386)	0.819		
Smoking (yes versus no)	2.488 (1.037–5.952)	0.039	4.132 (1.414–12.048)	0.009
Systolic BP	2.013 (0.922–4.394)	0.078	—	0.083
Diastolic BP	1.467 (0.677–3.178)	0.330		
Serum calcium	0.576 (0.264–1.255)	0.164	—	0.801
Serum phosphate	2.779 (1.256–6.149)	0.011	3.487 (1.34–9.090)	0.010
HDL cholesterol	1.535 (0.684–3.444)	0.298		
LDL cholesterol	0.433 (0.192–0.977)	0.042	—	0.148
Triglyceride	0.690 (0.308–1.546)	0.367		
Blood glucose	0.440 (0.198–0.980)	0.043	—	0.072
HbA1c	0.670 (0.292–1.534)	0.342		
C-reactive protein	0.369 (0.143–0.952)	0.036	—	0.390
AAC grade (versus grade 0)				
Grade 1	1.338 (0.4111–4.366)	0.627		
Grade 2	2.141 (0.700–6.535)	0.179	4.626 (1.241–17.244)	0.023
Grade 3	4.444 (1.353–14.705)	0.012	4.333 (1.134–16.551)	0.032

BP, blood pressure; HbA1c, hemoglobin A1c; HDL, high density lipoprotein; LDL, low density lipoprotein; rSO_2_, regional saturation of oxygen; AAC, aortic arch calcification; CI, confidence interval.
